# Comparative Effectiveness of Pharmacotherapies for the Risk of Attempted or Completed Suicide Among Persons With Borderline Personality Disorder

**DOI:** 10.1001/jamanetworkopen.2023.17130

**Published:** 2023-06-07

**Authors:** Johannes Lieslehto, Jari Tiihonen, Markku Lähteenvuo, Ellenor Mittendorfer-Rutz, Antti Tanskanen, Heidi Taipale

**Affiliations:** 1 Department of Forensic Psychiatry, University of Eastern Finland, Niuvanniemi Hospital, Kuopio, Finland; 2 Department of Clinical Neuroscience, Division of Insurance Medicine, Karolinska Institutet, Stockholm, Sweden; 3 Center for Psychiatry Research, Stockholm City Council, Stockholm, Sweden; 4 School of Pharmacy, University of Eastern Finland, Kuopio, Finland

## Abstract

**Importance:**

Suicidal behavior is a significant clinical concern in individuals with borderline personality disorder (BPD), but the effectiveness of pharmacotherapy on reducing suicide risk has remained unknown.

**Objective:**

To study the comparative effectiveness of different pharmacotherapies in preventing attempted or completed suicides in patients with BPD in Sweden.

**Design, Setting and Participants:**

In this comparative effectiveness research study, nationwide Swedish register databases of inpatient care, specialized outpatient care, sickness absences, and disability pensions were used to identify patients aged 16 to 65 years with registered treatment contact due to BPD during 2006 to 2021. Data were analyzed from September to December 2022. A within-individual design was used, in which each patient was used as their own control to eliminate selection bias. To control protopathic bias, sensitivity analyses were conducted, in which the first 1 or 2 months of medication exposure were omitted from the analyses.

**Main outcomes and Measures:**

Hazard ratio (HR) for attempted or completed suicide.

**Results:**

A total of 22 601 patients with BPD (3540 [15.7%] men; mean [SD] age, 29.2 [9.9] years) were included. During the 16-year follow-up (mean [SD] follow-up, 6.9 [5.1] years), 8513 hospitalizations due to attempted suicide and 316 completed suicides were observed. Attention-deficit/hyperactive disorder (ADHD) medication treatment, compared with its nonuse, was associated with a decrease in the risk of attempted or completed suicide (HR, 0.83; 95% CI, 0.73-0.95; false discovery rate [FDR]–corrected *P* = .001). Treatment with mood stabilizers did not have a statistically significant association with the main outcome (HR, 0.97; 95% CI, 0.87-1.08; FDR-corrected *P* = .99). Antidepressant (HR, 1.38; 95% CI, 1.25-1.53; FDR-corrected *P* < .001) and antipsychotic (HR, 1.18; 95% CI, 1.07-1.30; FDR-corrected *P* < .001) treatments were associated with an elevated risk of attempted or completed suicide. Of the investigated pharmacotherapies, treatment with benzodiazepines was associated with the highest risk of attempted or completed suicide (HR, 1.61; 95% CI, 1.45-1.78; FDR-corrected *P* < .001). These results remained similar when controlling for potential protopathic bias.

**Conclusions and Relevance:**

In this comparative effectiveness research study of a Swedish nationwide cohort, ADHD medication was the only pharmacological treatment associated with reduced risk of suicidal behavior among patients with BPD. Conversely, the findings suggest that benzodiazepines should be used with care among patients with BPD due to their association with increased risk of suicide.

## Key Points

**Question:**

What is the comparative effectiveness of pharmacological treatments on the risk of suicidal behavior among individuals with borderline personality disorder (BPD)?

**Findings:**

In this comparative effectiveness research study of 22 601 individuals with BPD, the use of attention-deficit/hyperactivity (ADHD) medication was associated with a reduced risk of suicidal behavior. No other pharmacotherapy (ie, antidepressants, antipsychotics, mood stabilizers, or benzodiazepines) was associated with a reduced risk of suicidal behavior.

**Meaning:**

These findings suggest that ADHD medication should be the preferred choice for individuals with BPD with ADHD symptoms and suicidal behavior.

## Teaser

This comparative effectiveness research study examines different pharmacotherapies’ association with the risk of attempted or completed suicide among patients with borderline personality disorder in Sweden.

## Introduction

Suicidal behavior is a significant clinical concern in individuals with borderline personality disorder (BPD). Suicidal ideation and attempts in individuals with BPD have lifetime prevalence estimates ranging from 84% to 94%,^1,2^ and approximately 5% to 10% of individuals with BPD eventually die by suicide.^3,4^ Furthermore, as many as 56% of suicidal patients admitted to a psychiatric hospital have BPD.^5^

Psychotherapy, particularly dialectical behavior therapy, has been shown to be effective in reducing suicidal behavior.^6^ However, partially because of poor access to psychotherapeutic treatments,^7^ pharmacotherapy is often preferred even despite a clear consensus on its role in clinical guidelines. Most individuals with BPD are treated with pharmacotherapy,^8,9^ but little is known about its effectiveness on suicidal behavior.

According to a recent Cochrane systematic review and meta-analysis of prior randomized clinical trials (RCTs), the evidence for the efficacy of pharmacotherapies in reducing suicide risk in BPD is inconclusive, and there are just a handful of small trials on this topic.^10^ Specifically, treatment with antipsychotics (standardized mean difference [SMD], 0.05; 95% CI, −0.18 to 0.29; 7 trials, 854 individuals), antidepressants (SMD, −0.26; 95% CI, −1.62 to 1.09; 2 trials, 45 individuals), mood stabilizers (SMD, −0.36; 95% CI, −1.96 to 1.25; 2 trials, 44 individuals), or benzodiazepines (SMD, 0.75; 95% CI, −0.18 to 1.68; 1 trial, 25 individuals) was not associated with a reduced risk of suicidal behavior. However, this could be because suicide is a rare occurrence during a brief follow-up in a typical RCT. Also, individuals with BPD with severe suicidal behavior are typically excluded from RCTs because of stringent exclusion criteria.

By using large, unselected nationwide electronic databases with long follow-ups, observational studies can overcome some of the issues present in typical RCTs, such as short follow-up periods and the exclusion of patients with severe comorbidities. Two large observational studies have investigated the effectiveness of antipsychotics on suicidal behavior in patients with personality disorders, including BPD. A self-controlled case series analysis with 1082 individuals (753 patients with BPD) in primary care in the United Kingdom found that rates of self-harm were reduced in the month after (vs before) the prescription of quetiapine.^11^ Nonetheless, patients demonstrated a high risk of suicidal behavior throughout the first year of quetiapine prescribing compared with a year before commencing. A nationwide Danish register study of 79 253 individuals (42 987 patients with BPD) on antipsychotic treatment found that rates of suicidal behavior were 32% lower during antipsychotic treatment compared with time without antipsychotic treatment.^12^ However, individuals with a comorbid psychotic disorder were not excluded from the study, which might have affected the findings, as treatment with antipsychotics in patients with schizophrenia is associated with a reduced risk of suicide.^13^

Altogether, the evidence for the effectiveness of various pharmacotherapy approaches in reducing suicidal behavior among patients with BPD remains inconsistent, with some types of medications showing promise in some patients with BPD while others have inconclusive results. To our knowledge, there are no extensive observational studies on the comparative effectiveness of antipsychotics in patients with BPD without other personality disorders and no studies on pharmacotherapies other than antipsychotics. Also, although more than one-third of individuals with BPD have comorbid attention-deficit/hyperactivity disorder (ADHD) symptoms,^14^ the association between ADHD medication treatment and suicidal behavior has remained unexplored, despite existing evidence suggesting these medications are associated with a decreased risk of suicide in patients with ADHD.^15,16^ Here, using nationwide Swedish register databases with up to 16 years of follow-up, we aimed to study the comparative effectiveness of several commonly used pharmacological treatments (ie, antipsychotics, antidepressants, mood stabilizers, benzodiazepines, and ADHD medications) on the risk of attempted or completed suicides among individuals with BPD.

## Methods

### Study Design and Data Acquisition

The Stockholm Regional Ethics Board approved the study project (decision Nos. 2007/762-3 and 2021-06441-02). As this study was conducted using registry-based data, there was no obligation for informed consent according to the Swedish legislation. We used a priori specification of the research question and analytical plans (including reporting of the results) in accordance with the International Society for Pharmacoeconomics and Outcomes Research (ISPOR) reporting guideline for comparative effectiveness research. We used Swedish electronic nationwide patient registries that encompass comprehensive data for individuals receiving public health care. To identify the study population, we used data from inpatient and specialized outpatient care from the National Patient Registry as well as data on sickness absence and disability pension from the Microdata for Analysis of Social Insurance (MiDAS) register. A detailed description of the used registries is provided in eMethods in Supplement 1. The extensive population-based public health care system in Sweden has rendered nationwide registries invaluable resources for conducting a multitude of epidemiological investigations.

Residents of Sweden between the ages of 16 and 65 years having a registered treatment contact for BPD (*International Statistical Classification of Diseases and Related Health Problems, Tenth Revision *[ICD-10] code F60.3) between January 1, 2006, and June 30, 2021, were included in the study. The Swedish National Patient Registry has been validated for the diagnosis of BPD using a retrospective review of patient records.^17^ The follow-up started at the first diagnosis or January 1, 2006, for those diagnosed before that (the diagnostic data are available since 1997). Individuals with comorbid nonaffective psychotic disorders (*ICD-10* codes F20-F29), bipolar disorder (*ICD-10* code F31), psychotic depression (*ICD-10* code F32.3), and personality disorders other than BPD (*ICD-10* codes F60-61 except F60.3) were excluded. The analyses were also censored to these diagnoses, in addition to death, emigration, and end of data linkage (June 30, 2021).

### Exposure

Our primary exposure was group-level medication treatment (eg, antidepressant treatment). In addition, we investigated exposure at the level of specific agents (eg, treatment with quetiapine), which was a secondary exposure. The reference for each treatment was the nonuse of that medication group (eg, citalopram was compared with the nonuse of antidepressants). We categorized treatment exposure as follows: antipsychotics (Anatomical Therapeutic Chemical [ATC] code N05A, excluding lithium [N05AN01]), antidepressants (ATC code N06A), mood stabilizers (ATC codes N03AF, N03AG, N03AX, and N05AN01), benzodiazepines and similar compounds (ATC codes N05BA, N05CD, and N05CF), and ADHD medications (ATC code N06BA). We derived the drug usage periods from prescription drug purchases using the Prescriptions to Drug Use Periods (PRE2DUP) method, detailed elsewhere.^18^ The PRE2DUP method is based on the calculation of sliding averages of defined daily dosages, the amounts of drugs purchased, and individual drug use patterns. Hospital stays and medicine stockpiling are also incorporated into the model.

### Outcomes

Our primary outcome was attempted suicide (ie, hospitalization) or completed suicide (ie, death), and our secondary outcome was completed suicide. The diagnoses for the outcome were extracted from the inpatient care, and causes of death diagnoses (*ICD-10* codes X60-84) were reinforced with diagnoses with undetermined intent (*ICD-10 *codes Y10-34) to compensate for underreporting of suicide attempts. Pharmacotherapy in BPD is often initiated during high-risk suicidal behavior, which could induce more negative associations between a given treatment and the outcome due to lag until the treatment reaches its full efficacy. To control for this well-reported protopathic bias,^19^ we ran sensitivity analyses in which the first 1 or 2 months of medication exposure were omitted from the follow-up.^20^

### Statistical Analysis

We used SAS version 9.4 (SAS Institute) for the statistical analyses. To control for the potential selection bias caused by the nonrandomization of pharmacotherapies, we used a within-individual Cox regression model in which each patient is his or her own control. A detailed description is provided elsewhere^21^ and in eFigure 1 in Supplement 1. Our primary outcome was modeled as recurring occurrences. We adjusted these models to account for the temporal order of treatments, the period since cohort entry, and the use of concomitant psychotropic drugs, such as antidepressants, benzodiazepines and related pharmaceuticals, mood stabilizers, ADHD medications, and medications used to treat substance use disorders (disulfiram, acamprosate, naltrexone, nalmefene, buprenorphine, and methadone). In the within-individual analysis, the follow-up time was reset to zero after each outcome event to compare treatment durations within an individual.^20^

We also performed traditional between-individual analyses (multivariate-adjusted Cox regression model, adjusted for gender, age, and educational level at cohort entry; the number of previous hospitalizations due to suicide attempts; time from first BPD diagnosis; and other concomitant psychiatric medications) to analyze the secondary outcome since completed suicide is a one-time event. Also, between-individual analyses were conducted for the primary outcome to investigate whether the results from between-individual analyses correspond to within-individual analyses.

We presented the results using hazard ratios (HRs) and 95% CIs. In each medication group analysis (ie, primary exposure), we used the Benjamini-Hochberg false discovery rate (FDR) correction to control false positives caused by multiple comparisons.^22^ We set the statistical significance to FDR-corrected *P* < .05. However, due to the low number of events, we present uncorrected *P* values for individual pharmacotherapies (ie, secondary exposure). We conducted our data analysis from September to December 2022.

## Results

### Sociodemographic and Clinical Characteristics

Sociodemographic and clinical data are presented in the Table. We collected data on 22 601 patients with BPD with an mean (SD) baseline age of 29.2 (9.9) years who were followed up from 2006 to 2021 (mean [SD] follow-up, 6.9 [5.1] years). Most patients were women (19 061 [84.3%]; 3540 [15.7%] men), Swedish-born (19 809 [87.7%]), and single without children (13 032 [57.7%]). In the total cohort, 9525 individuals (42.1%) had any income from work at baseline, and 2844 (12.6%) were granted a disability pension at cohort entry. One-third of the patients (7626 [33.7%]) had comorbid substance use disorder; 12 801 (56.6%), depression; 16 125 (71.4%), anxiety disorder; and 3885 (17.2%), ADHD at baseline. Also, one-third of the sample (7330 [32.4%]) had attempted suicide previously.

**Table.** Characteristics of the Nationwide Study Population (N = 22 601) at Cohort Entry

Characteristic	Individuals, No. (%) (N = 22 601)
Age, mean (SD), y	29.2 (9.9)
Gender	
Men	3540 (15.7)
Women	19 061 (84.3)
Country of birth	
Sweden	19 809 (87.7)
Other European country	1213 (5.4)
Rest of the world	1579 (7.0)
Education	
<9 y	7695 (34.1)
10-12 y	9852 (43.6)
>12 y	4395 (19.5)
Missing	659 (2.9)
Family situation	
Married without children	625 (2.8)
Married with children	2288 (10.1)
Single without children	13 032 (57.7)
Single with children	2417 (10.7)
Aged <20 y and living at home	4037 (17.9)
Missing information	202 (0.9)
Income	
Any income from work (year before cohort entry)	9525 (42.1)
Unemployment during previous year	
1-180 d	4336 (19.2)
>180 d	757 (3.4)
No unemployment	17 508 (77.5)
On disability pension at cohort entry	2844 (12.6)
Sickness absence during a year before cohort entry	
1-90 d	2446 (10.8)
>90 d	3100 (13.7)
No sickness absence	17 055 (75.5)
Comorbidities	
Any substance use disorder	7626 (33.7)
Depression	12 801 (56.6)
Anxiety disorder	16 125 (71.4)
ADHD	3885 (17.2)
Previous suicide attempt	7330 (32.4)
Cardiovascular disease	1908 (8.4)
Previous cancer	258 (1.1)
Diabetes	451 (2.0)
Asthma	1951 (8.6)
Use of specific medications at any time after cohort entry	
Antidepressants	18 411 (81.5)
Antipsychotics	9283 (41.1)
Mood stabilizers	7165 (31.7)
Benzodiazepines and related drugs	12 660 (56.0)
ADHD medications	5520 (24.4)
Substance use disorder medications	2381 (10.5)

Abbreviation: ADHD, attention-deficit/hyperactivity disorder.

At some point during the follow-up, most patients were treated with antidepressants (18 411 [81.5%]), of which sertraline was the most commonly used treatment (6479 [28.7%]). Also, more than half of the patients were receiving benzodiazepines (12 660 [56.0%]) at some point during follow-up. In addition, 9283 patients (41.1%) were treated with antipsychotics, 7165 (31.7%) with mood stabilizers, and 5520 (24.4%) with ADHD medications. Of these treatments, the most commonly used antipsychotic was quetiapine (5466 [24.2%]), the most commonly used mood stabilizer was lamotrigine (5415 [24.0%]), and the most commonly used ADHD medication was methylphenidate (4256 [18.8%]). The percentages of individuals using each medication during follow-up are presented in eTable 1 in Supplement 1.

Over follow-up, we observed 8513 hospitalizations due to attempted suicide and 316 completed suicides. The median (IQR) time from the diagnosis of BPD to the first hospitalization due to attempted suicide was 271 (55-895) days and to completed suicide, 1300 (412-2605) days.

### Risk of Attempted or Completed Suicide

**Figure 1.**
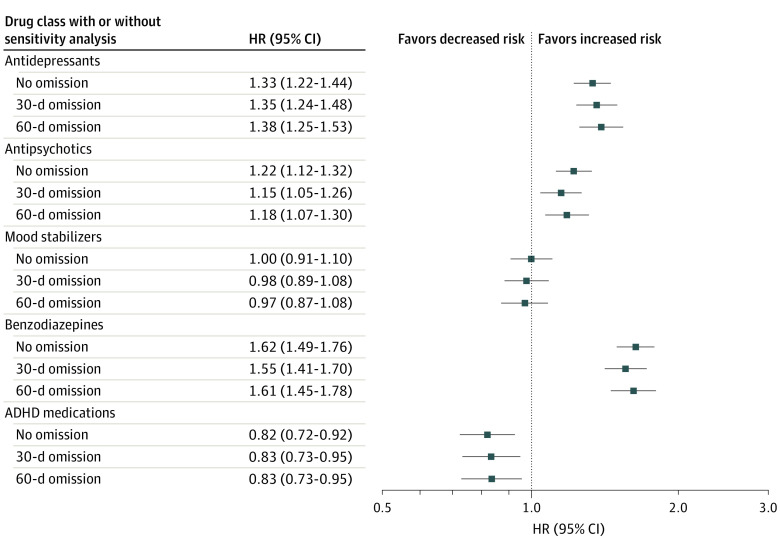
Risk of Attempted or Completed Suicide During Use of Pharmacotherapy Compared With Nonuse of the Medication Class in Within-Individual Analyses Sensitivity analyses excluding first 30 and 60 days of medication exposure are also presented. ADHD indicates attention-deficit/hyperactivity disorder; HR, hazard ratio.

The associations between pharmacotherapeutic treatments and attempted or completed suicides are depicted in Figure 1. Treatment with ADHD medication was associated with a decreased risk of attempted or completed suicide (HR, 0.82; 95% CI, 0.72-0.92; FDR-corrected *P* = .001). Mood stabilizer treatment was not associated with the main outcome (HR, 1.00; 95% CI, 0.91-1.10; FDR-corrected *P* = .99). Treatment with benzodiazepines (HR, 1.62; 95% CI, 1.49-1.76; FDR-corrected *P*< .001), antidepressants (HR, 1.33; 95% CI, 1.22-1.44; FDR-corrected *P* < .001), and antipsychotics (HR, 1.22; 95% CI, 1.12-1.32; FDR-corrected *P* < .001) was associated with an increased risk of attempted or completed suicides. As shown in Figure 1, these findings remained basically the same even when omitting the first 30 or 60 days of each medication exposure period.

**Figure 2.**
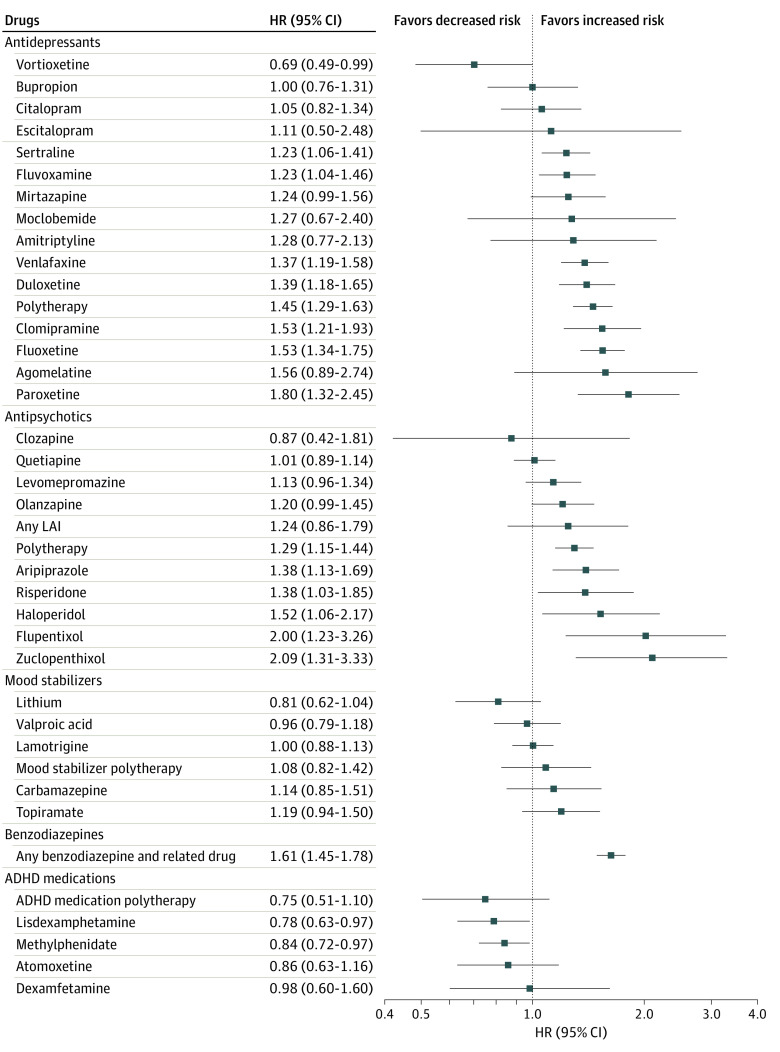
Risk of Attempted or Completed Suicide During Use of Individual Pharmacotherapeutic Agents Compared With Nonuse of the Medication Class in Within-Individual Analyses ADHD indicates attention-deficit/hyperactivity disorder; HR, hazard ratio; LAI, long-acting injectable.

The associations between specific pharmacotherapies and attempted or completed suicide are depicted in Figure 2 and eTable 2 in Supplement 1. Treatment with vortioxetine (HR, 0.69; 95% CI, 0.49-0.99; *P* = .04), lisdexamphetamine (HR, 0.78; 95% CI, 0.63-0.97; *P* = .03), and methylphenidate (HR, 0.84; 95% CI, 0.72-0.97; *P* = .02) was associated with a lower risk of attempted or completed suicide. We did not find statistically significant associations between the investigated mood stabilizers and the primary outcome. Of all the explored antidepressant medications, we discovered that paroxetine was associated with the highest risk of attempted or completed suicide (HR, 1.80; 95% CI, 1.32-2.45; *P* < .001). Of all the explored antipsychotic treatments, zuclopenthixol was associated with the highest risk of attempted or completed suicide (HR, 2.09; 95% CI, 1.31-3.33; *P* = .002).

Traditional between-individual analysis aligned with the findings already described (eTable 3 and eFigure 2 in Supplement 1). For the primary outcome, attempted or completed suicide, the rank order of HRs from within-individual analyses was quite similar to that of HRs from between-individual analyses (Spearman ρ = 0.67).

### Risk of Completed Suicide

**Figure 3.**
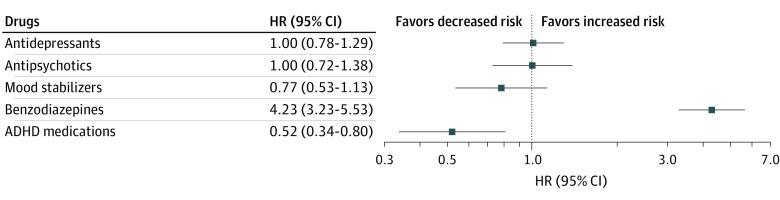
Risk of Completed Suicide During Use of Pharmacotherapy Compared With Nonuse of the Medication Class in Between-Individual Analyses ADHD indicates attention-deficit/hyperactivity disorder; HR, hazard ratio.

The associations between pharmacological treatments and the secondary outcome are presented in Figure 3. During follow-up, the use of ADHD medication was associated with a decreased risk of completed suicide (HR, 0.52; 95% CI, 0.34-0.80; FDR-corrected *P* = .008). The use of mood stabilizers (HR, 0.77; 95% CI, 0.53-1.13; FDR-corrected *P* = .30), antidepressants (HR, 1.00; 95% CI, 0.78-1.29; FDR-corrected *P* = .99), or antipsychotics (HR, 1.00; 95% CI, 0.72-1.38; FDR-corrected *P* = .99) was not associated with suicide completion. Benzodiazepine treatment was associated with a high risk of completed suicide (HR, 4.23; 95% CI, 3.23-5.53; FDR-corrected *P* <.001).

## Discussion

To our knowledge, this is the first study to explore the comparative effectiveness of pharmacotherapies on suicidal behavior in individuals with BPD. Our study adds to the literature by providing insight concerning the use of pharmacotherapy and suicide risk, which is a major clinical challenge for treating BPD. Our main findings indicate that, compared with individuals’ nonuse periods, ADHD medication was associated with a reduced risk of attempted or completed suicide. None of the other investigated pharmacotherapies (ie, antipsychotics, antidepressants, mood stabilizers, and benzodiazepines) were associated with favorable outcomes. In fact, treatment with benzodiazepines was consistently associated with an increased risk of attempted and completed suicide.

Although there has been some concern that ADHD medications may exacerbate suicidal thoughts and behaviors,^16^ large epidemiological studies have reported that the use of these medications is associated with a low risk of suicidal behavior in ADHD.^15,23^ According to our results, patients with BPD are at their lowest risk for suicidal behavior when treated (vs not treated) with ADHD medications, particularly stimulant compounds. The lowest risk of attempted or completed suicide was detected for lisdexamphetamine. Since the main clinical indication for these pharmacotherapies is ADHD, patients with BPD who are receiving ADHD medication treatment likely have comorbid ADHD symptoms. In fact, BPD and ADHD share symptoms, such as impulsivity and emotion dysregulation,^24,25^ thus providing potential overlapping target symptoms for pharmacotherapy in these 2 patient groups. Meta-analytical evidence indicates that treatment with ADHD medications is associated with decreased impulsivity,^26^ which is critical given that impulsivity is one of the strongest predictors of suicidal behavior in BPD.^27^ Strikingly, our findings suggest that up to a 48% decrease in the probability of suicide completion in patients with BPD is attributable to treatment with ADHD medication. Taken together, our findings indicate that ADHD medication should be the preferred choice for patients with BPD with ADHD symptoms and suicidal behavior.

Patients with BPD are frequently treated with benzodiazepines^8,28^ despite well-recognized impulsivity-related issues in these patients.^29^ Here, in our nationwide cohort of patients with BPD, more than half of the sample was treated with benzodiazepines at some point during the follow-up. During benzodiazepine treatment, the risk of attempted or completed suicide was increased compared with time without benzodiazepine treatment. Alarmingly, treatment with benzodiazepines was related to a 4-fold risk increment in suicide completion in patients with BPD. Our findings potentially result from well-reported augmented impulsivity and aggression among patients with BPD during benzodiazepine treatment^30,31^ that may facilitate suicidal behavior.

Despite the paucity of evidence, antidepressants, antipsychotics, and mood stabilizers are routinely used in BPD with the intention of treating suicidal behavior along with symptoms such as mood lability, anger, and impulsivity. Notably, 80% of our sample was treated with antidepressants, even though just a handful of RCTs have ever been undertaken on its usage in BPD.^10^ In the present study, using any of these pharmacotherapies was not associated with a reduced risk of attempted or completed suicide. Although antipsychotic and antidepressant treatment were related to a somewhat higher risk of attempted or completed suicide, the analysis on completed suicide did not indicate an elevated risk of suicide completion during treatment (vs nontreatment) periods. Altogether, our data suggest that treatment with antidepressants, antipsychotics, or mood stabilizers does not appear to reduce suicidal behavior in patients with BPD.

Previous work has shown relatively good alignment between the results from observational studies and placebo-controlled RCTs.^32^ Nevertheless, observational data has several limitations compared with placebo-controlled RCTs. First, the lack of randomization of pharmacotherapies in observational studies is a major issue.^19^ However, the present study’s use of within-individual modeling on actually purchased medications minimizes selection bias since each patient serves as his or her own control.^20^ Specifically, within-individual modeling eliminates time-invariant factors (eg, genetic differences) and reduces the impact of BPD severity and comorbidities.^20^ In within-individual design, the model needs to be only adjusted for time-varying factors, such as time since onset of illness, the temporal order of treatments, and other concomitant pharmacotherapy.

However, time-dependent factors are not addressed in within-individual analysis, which makes our results susceptible to protopathic bias.^19^ In particular, because pharmacological treatments for BPD frequently commence during abrupt crises, our findings may underestimate the potential therapeutic benefit of pharmacotherapies that had not yet reached their maximum efficacy before suicidal behavior. To address protopathic bias, we performed sensitivity analyses, and regardless of 30 or even 60 days of omission from the start of pharmacotherapy, the results were nearly identical to the primary analyses. While our sensitivity analysis may not have wholly eliminated protopathic bias, it is remarkable that the use of ADHD medications was still associated with a reduction in attempted or completed suicides. In contrast, the use of benzodiazepines was consistently associated with a substantial increase in suicide risk.

### Strengths and Limitations

The present study has both strengths and limitations. Compared with RCTs with typically small sample sizes, short follow-up periods, and strict exclusion criteria for suicidal behavior, the main strength of the present study is the utilization of unselected nationwide sample of more than 20 000 patients with BPD with up to 16 years follow-up. Consequently, our findings are likely to generalize to clinical practice. However, we lacked specific clinical parameters, such as the severity of BPD symptoms and indications for pharmacotherapy, that may have significance for suicidal behavior. Also, we lacked information regarding concomitant psychotherapy treatments, such as dialectical behavioral therapy, that have been shown to be effective in reducing suicidal behavior.^6^ Additionally, the study took place in a high-income country with a predominantly White population and a government-funded health care system offering access to health care to all residents with minimum costs for the patients. The findings may not be generalizable in other settings.

## Conclusions

In this comparative effectiveness research study of an unselected nationwide sample of patients with BPD, the use of ADHD medications, potentially due to diminished impulsivity, was consistently associated with a reduced risk of suicide. However, the use of antidepressants, antipsychotics, or mood stabilizers was not associated with a reduced risk of suicidality in BPD, even when potential protopathic bias was controlled. Lastly, benzodiazepine use was associated with a marked increment in suicide risk.

## Notes

Supplement 1.
eMethods.
**eTable 1.** Medications Used by the Persons With Borderline Personality Disorder at Some Point During the Follow-up **eTable 2.** The Association Between Use vs Nonuse of Medications and Risk of Attempted or Completed Suicide in Within-Individual Analysis **eTable 3.** The Association Between Use vs Nonuse of Medications and Risk of Attempted or Completed Suicide in Between-Individual Analysis **eFigure 1.** Study Design **eFigure 2.** Associations Between the Results From Within-Individual Modeling and Between-Individual Modeling

Supplement 2.
Data Sharing Statement
