# Supplementary material for: Comparative Effectiveness of Pharmacotherapies for the Risk of Attempted or Completed Suicide Among Persons With Borderline Personality Disorder
Source: JAMA Netw Open. 2023 Jun 7;6(6):e2317130. doi: 10.1001/jamanetworkopen.2023.17130 (PMC10248738; doi:10.1001/jamanetworkopen.2023.17130)
Supplement: Supplement 1. — eMethods. eTable 1. Medications Used by the Persons With Borderline Personality Disorder at Some Point During the Follow-up eTable 2. The Association Between Use vs Nonuse of Medications and Risk of Attempted or Completed Suicide in Within-Individual Analysis eTable 3. The Association Between Use vs Nonuse of Medications and Risk of Attempted or Completed Suicide in Between-Individual Analysis eFigure 1. Study Design eFigure 2. Associations Between the Results From Within-Individual Modeling and Between-Individual Modeling [file jamanetwopen-e2317130-s001.pdf]

Supplemental Online Content

Lieslehto J, Tiihonen J, Lähteenvuo M, Mittendorfer-Rutz E, Tanskanen A, Taipale H. Comparative effectiveness of pharmacotherapies for the risk of attempted or completed suicide among persons with borderline personality disorder. *JAMA Netw Open.* 2023;6(6):e2317130. doi:10.1001/jamanetworkopen.2023.17130

eMethods.

eTable 1. Medications Used by the Persons With Borderline Personality Disorder at Some Point During the Follow-up

eTable 2. The Association Between Use vs Nonuse of Medications and Risk of Attempted or Completed Suicide in Within-Individual Analysis

eTable 3. The Association Between Use vs Nonuse of Medications and Risk of Attempted or Completed Suicide in Between-Individual Analysis

eFigure 1. Study Design

eFigure 2. Associations Between the Results From Within-Individual Modeling and Between-Individual Modeling

This supplemental material has been provided by the authors to give readers additional information about their work.

© 2023 Lieslehto J et al. *JAMA Network Open.*

eMethods.

We identified the study population using data from inpatient and specialized outpatient care from the National Patient Registry as well as data on sickness absence and disability pension from the MiDAS register (Microdata for analysis of social insurance). The Prescribed Drug Register contained information about drugs dispensed. The classification of drug dispensing data is based on the anatomical therapeutic chemical (ATC) categorization, in addition to information on defined daily doses (DDDs), drug packages, and formulation. The Causes of Death Register was examined for death dates and causes. The longitudinal Integration Database for Health Insurance and Labor Market Studies (LISA) Register was used to collect information regarding the cohort's demographic characteristics and emigration.

eTable 1. Medications Used by the Persons With Borderline Personality Disorder (N= 22,601) at Some Point During the Follow-up

Treatment	Medication group	Number of Users	Percentage
Polytherapy	Antidepressant	9259	40.97
Sertraline	Antidepressant	6479	28.67
Fluoxetine	Antidepressant	4463	19.75
Fluvoxamine	Antidepressant	4204	18.60
Venlafaxine	Antidepressant	3986	17.64
Mirtazapine	Antidepressant	3470	15.35
Duloxetine	Antidepressant	2862	12.66
Bupropion	Antidepressant	2741	12.13
Citalopram	Antidepressant	2384	10.55
Amitriptyline	Antidepressant	1525	6.75
Vortioxetine	Antidepressant	1032	4.57
Paroxetine	Antidepressant	802	3.55
Clomipramine	Antidepressant	580	2.57
Agomelatine	Antidepressant	523	2.31
Moclobemide	Antidepressant	338	1.50
Escitalopram	Antidepressant	103	0.46
Quetiapine	Antipsychotic	5466	24.18
Polytherapy	Antipsychotic	2679	11.85
Olanzapine	Antipsychotic	2337	10.34
Levomepromazine	Antipsychotic	1696	7.50
Aripiprazole	Antipsychotic	1587	7.02
Risperidone	Antipsychotic	858	3.80
Flupentixol	Antipsychotic	521	2.31
Haloperidol	Antipsychotic	238	1.05
Zuclopenthixol	Antipsychotic	144	0.64
Any LAI	Antipsychotic	71	0.31
Clozapine	Antipsychotic	36	0.16
Lamotrigine	Mood Stabilizer	5415	23.96
Valproic acid	Mood Stabilizer	1076	4.76
Topiramate	Mood Stabilizer	678	3.00
Polytherapy	Mood Stabilizer	666	2.95
Carbamazepine	Mood Stabilizer	653	2.89
Lithium	Mood Stabilizer	513	2.27
Methylphenidate	ADHD Medication	4256	18.83
Lisdexamphetamine	ADHD Medication	2580	11.42
Polytherapy	ADHD Medication	1927	8.53
Atomoxetine	ADHD Medication	1125	4.98
Dexamfetamine	ADHD Medication	459	2.03

Abbreviations: LAI=Long-Acting Injectable

© 2023 Lieslehto J et al. *JAMA Network Open.*

eTable 2. The Association Between Use vs Nonuse of Medications and Risk of Attempted or Completed Suicide in Within-Individual Analysis

Treatment	Medication group	HR	95%CI	P-value
Vortioxetine	Antidepressant	0.69	0.49-0.99	0.044
Bupropion	Antidepressant	1.00	0.76-1.31	0.978
Citalopram	Antidepressant	1.05	0.82-1.34	0.688
Escitalopram	Antidepressant	1.11	0.50-2.48	0.792
Sertraline	Antidepressant	1.23	1.06-1.41	0.006
Fluvoxamine	Antidepressant	1.23	1.04-1.46	0.017
Mirtazapine	Antidepressant	1.24	0.99-1.56	0.062
Moclobemide	Antidepressant	1.27	0.67-2.40	0.468
Amitriptyline	Antidepressant	1.28	0.77-2.13	0.339
Venlafaxine	Antidepressant	1.37	1.19-1.58	<0.001
Duloxetine	Antidepressant	1.39	1.18-1.65	<0.001
Polytherapy	Antidepressant	1.45	1.29-1.63	<0.001
Clomipramine	Antidepressant	1.53	1.21-1.93	<0.001
Fluoxetine	Antidepressant	1.53	1.34-1.75	<0.001
Agomelatine	Antidepressant	1.56	0.89-2.74	0.120
Paroxetine	Antidepressant	1.80	1.32-2.45	<0.001
Clozapine	Antipsychotic	0.87	0.42-1.81	0.712
Quetiapine	Antipsychotic	1.01	0.89-1.14	0.889
Levomepromazine	Antipsychotic	1.13	0.96-1.34	0.153
Olanzapine	Antipsychotic	1.20	0.99-1.45	0.061
Any LAI	Antipsychotic	1.24	0.86-1.79	0.252
Polytherapy	Antipsychotic	1.29	1.15-1.44	<0.001
Risperidone	Antipsychotic	1.38	1.03-1.85	0.029
Aripiprazole	Antipsychotic	1.38	1.13-1.69	0.002
Haloperidol	Antipsychotic	1.52	1.06-2.17	0.022
Flupentixol	Antipsychotic	2.00	1.23-3.26	0.006
Zuclopenthixol	Antipsychotic	2.09	1.31-3.33	0.002
Lithium	Mood Stabilizer	0.81	0.62-1.04	0.101
Valproic acid	Mood Stabilizer	0.96	0.79-1.18	0.713
Lamotrigine	Mood Stabilizer	1.00	0.88-1.13	0.960
Polytherapy	Mood Stabilizer	1.08	0.82-1.42	0.582
Carbamazepine	Mood Stabilizer	1.14	0.85-1.51	0.386
Topiramate	Mood Stabilizer	1.19	0.94-1.50	0.152
Polytherapy	ADHD Medication	0.75	0.51-1.10	0.139
Lisdexamphetamine	ADHD Medication	0.78	0.63-0.97	0.027
Methylphenidate	ADHD Medication	0.84	0.72-0.97	0.021
Atomoxetine	ADHD Medication	0.86	0.63-1.16	0.317
Dexamfetamine	ADHD Medication	0.98	0.60-1.60	0.934

Abbreviations: HR=Hazard Ratio, 95% CI=95% Confidence Interval and LAI=Long-Acting Injectable

© 2023 Lieslehto J et al. *JAMA Network Open.*

eTable 3. The Association Between Use vs Nonuse of Medications and Risk of Attempted or Completed Suicide in Between-Individual Analysis

Treatment	Medication group	HR	95%CI	P-value
Amitriptyline	Antidepressant	0.97	0.70-1.33	0.840
Bupropion	Antidepressant	0.99	0.79-1.22	0.889
Moclobemide	Antidepressant	1.09	0.65-1.83	0.757
Mirtazapine	Antidepressant	1.09	0.94-1.27	0.252
Escitalopram	Antidepressant	1.21	0.50-2.91	0.674
Citalopram	Antidepressant	1.25	1.07-1.46	0.006
Fluvoxamine	Antidepressant	1.28	1.14-1.44	<0.001
Agomelatine	Antidepressant	1.30	0.94-1.79	0.114
Venlafaxine	Antidepressant	1.32	1.19-1.47	<0.001
Clomipramine	Antidepressant	1.40	1.18-1.66	<0.001
Polytherapy	Antidepressant	1.41	1.26-1.57	<0.001
Sertraline	Antidepressant	1.45	1.24-1.69	<0.001
Vortioxetine	Antidepressant	1.56	0.97-2.51	0.068
Duloxetine	Antidepressant	1.57	1.28-1.92	<0.001
Fluoxetine	Antidepressant	1.58	1.42-1.76	<0.001
Paroxetine	Antidepressant	1.79	1.06-3.02	0.029
Clozapine	Antipsychotic	1.12	0.68-1.84	0.665
Risperidone	Antipsychotic	1.20	0.99-1.45	0.064
Quetiapine	Antipsychotic	1.20	1.06-1.35	0.004
Flupentixol	Antipsychotic	1.24	0.95-1.62	0.107
Levomepromazine	Antipsychotic	1.25	1.08-1.45	0.003
Olanzapine	Antipsychotic	1.43	1.25-1.63	<0.001
Aripiprazole	Antipsychotic	1.47	1.27-1.69	<0.001
Haloperidol	Antipsychotic	1.64	1.27-2.10	<0.001
Any LAI	Antipsychotic	1.67	1.16-2.39	0.006
Polytherapy	Antipsychotic	1.82	1.58-2.11	<0.001
Zuclopenthixol	Antipsychotic	1.87	1.29-2.70	0.001
Lithium	Mood Stabilizer	0.96	0.75-1.23	0.744
Carbamazepine	Mood Stabilizer	0.98	0.81-1.19	0.869
Lamotrigine	Mood Stabilizer	0.99	0.88-1.11	0.802
Polytherapy	Mood Stabilizer	1.06	0.83-1.35	0.643
Topiramate	Mood Stabilizer	1.23	0.97-1.56	0.084
Valproic acid	Mood Stabilizer	1.24	0.86-1.78	0.255
Methylphenidate	ADHD Medication	0.89	0.79-1.00	0.057
Lisdexamphetamine	ADHD Medication	0.90	0.76-1.06	0.202
Polytherapy	ADHD Medication	0.94	0.67-1.34	0.744
Atomoxetine	ADHD Medication	0.95	0.76-1.19	0.659
Dexamfetamine	ADHD Medication	1.01	0.41-2.49	0.989

Abbreviations: HR=Hazard Ratio, 95% CI=95% Confidence Interval and LAI=Long-Acting Injectable

© 2023 Lieslehto J et al. *JAMA Network Open.*

eFigure 1. Study Design

A **Illustration how comparisons are constructed in within-individual design (within a drug class)**

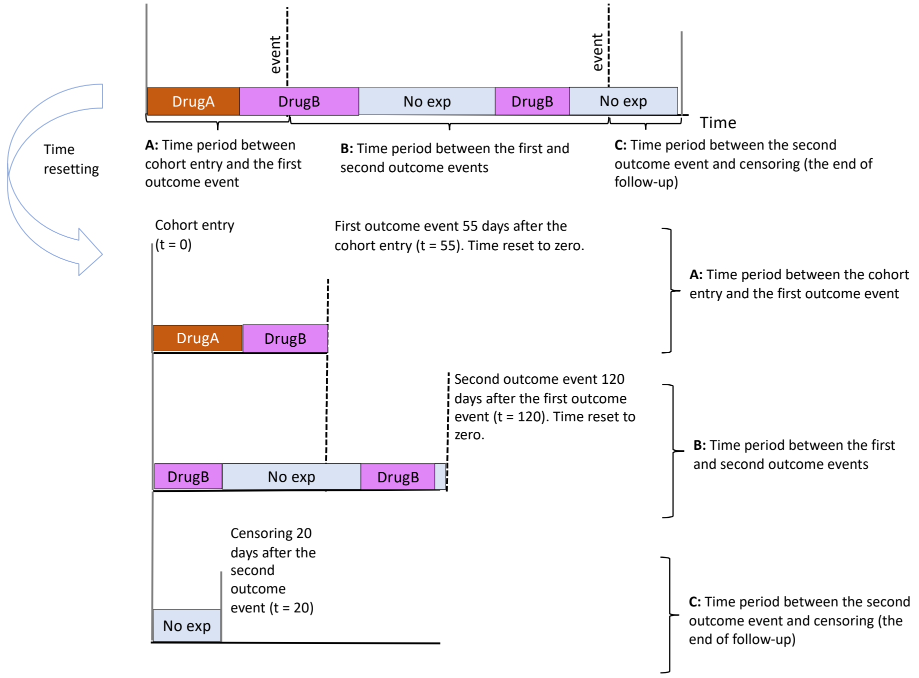

B **Concomitant psychotropic medications are adjusted for in the analyses (on drug-class level in this simplified example)**

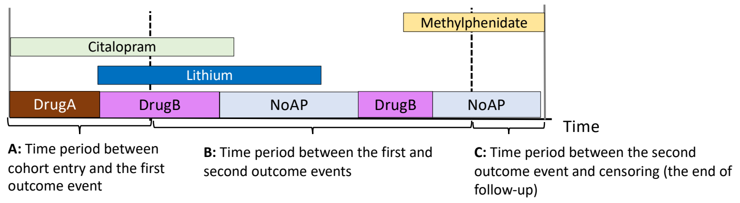

Analyses dataset structure for the above drug use history

row#	ID	start	stop	AP	MS	AD	ADHD	series	censor	init	end
1	3	1	25	DrugA	0	Cit	0	1	0	1	25
2	3	26	55	DrugB	Li	Cit	0	1	1	26	55
3	3	56	82	DrugB	Li	Cit	0	2	0	1	27
4	3	83	90	NoAP	Li	Cit	0	2	0	28	35
5	3	91	125	NoAP	Li	0	0	2	0	36	70
6	3	126	133	NoAP	0	0	0	2	0	71	78
7	3	134	155	DrugB	0	0	0	2	0	79	100
8	3	156	167	DrugB	0	0	MPh	2	0	101	112
9	3	168	175	NoAP	0	0	MPh	2	1	113	120
10	3	176	196	NoAP	0	0	MPh	3	0	1	20

start and ***stop*** describe continuous time since cohort entry (start=1) until the end of follow-up (stop=196) as days, and they form the basis for the between-individual model.
AP=antipsychotic describes the time-varying use of two specific antipsychotics (DrugA and DrugB) and non-use of antipsychotics (NoAP).
MS (mood stabilizer), ***AD*** (antidepressant), and ***ADHD*** (drugs for the treatment of ADHD) describe time-varying use of these medications on drug level (Li=lithium, Cit=citalopram, and MPH=methylphenidate as examples).
series indicates which rows belong to the same series of within-individual models (time is reset to zero in ***init*** and ***end*** variables when the series changes).
censor indicates whether the period ended at the outcome event (censor=1) or censoring (censor=0). After ***censor***=1, time is reset, and the next row starts with ***init***=1.
init and ***end*** assign time variables for the within-individual model (where time is reset to zero after each outcome event).
Row in the analyses dataset changes every time there is a change in exposures (AP, MS, AD, or ADHD) or outcome event happens.

A) In the within-individual design, each individual forms his/her own stratum. Within each stratum, time is reset after each outcome event. Use of different specific drugs (antipsychotics in this example, DrugA and DrugB in this example) vs. non-use of (“No exp”) are treated as time-varying exposures in the stratified Cox model. After resetting, time periods from the same individual are used in comparisons in the same way as different individuals are compared in a traditional Cox model (in the case of time-varying exposure). The method is described in: Paul D Allison: Fixed Effects Regression Models, SAGE Publications, 2009. **B)** Illustration how concomitant psychotropic medications are taken into account in the analyses.

© 2023 Lieslehto J et al. *JAMA Network Open.*

eFigure 2. Associations Between the Results From Within-Individual Modeling and Between-Individual Modeling

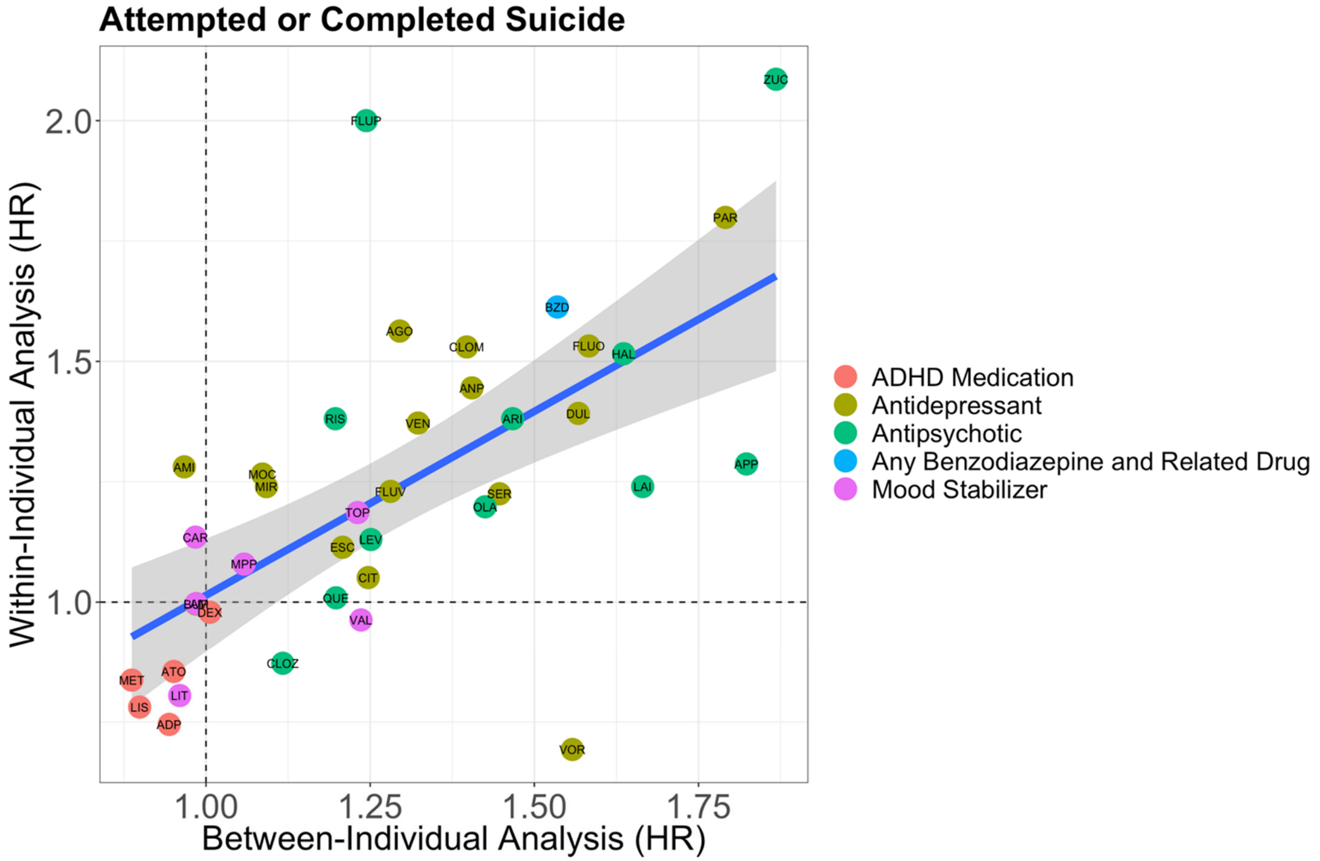

Spearman's rho=0.674, Pearson correlation=0.642. Dashed lines represent Hazard Ratio=1.0. Shaded grey represents 95%CI. Abbreviations: ADP=ADHD medication polytherapy, AGO=Agomelatine, AMI=Amitriptyline, ANP=Antidepressant polytherapy, APP=AP Polytherapy, ARI=Aripiprazole, ATO=Atomoxetine, BUP=Bupropion, BZD=Any benzodiazepine and related drug, CAR=Carbamazepine, CIT=Citalopram, CLOM=Clomipramine, CLOZ=Clozapine, DEX=Dexamfetamine, DUL=Duloxetine, ESC=Escitalopram, FLUO=Fluoxetine, FLUP=Flupentixol, FLUV=Fluvoxamine, HAL=Haloperidol, LAI=Any LAI, LAM=Lamotrigine, LEV=Levomepromazine, LIS=Lisdexamphetamine, LIT=Lithium, MET=Methylphenidate, MIR=Mirtazapine, MOC=Moclobemide, MPP=Mood stabilizer polytherapy, OLA=Olanzapine, PAR=Paroxetine, QUE=Quetiapine, RIS=Risperidone, SER=Sertraline, TOP=Topiramate, VAL=Valproic acid, VEN=Venlafaxine, VOR=Vortioxetine, ZUC=Zuclopenthixol.

© 2023 Lieslehto J et al. *JAMA Network Open.*
