# Supplementary material for: Comparative Effectiveness of Pharmacotherapies for the Risk of Attempted or Completed Suicide Among Persons With Borderline Personality Disorder
Source: JAMA Netw Open. 2023 Jun 7;6(6):e2317130. doi: 10.1001/jamanetworkopen.2023.17130 (PMC10248738; doi:10.1001/jamanetworkopen.2023.17130)
Supplement: Supplement 2. — Data Sharing Statement [file jamanetwopen-e2317130-s002.pdf]

Data Sharing Statement

Lieslehto. Comparative Effectiveness of Pharmacotherapies for the Risk of Attempted or Completed Suicide Among Persons With Borderline Personality Disorder. *JAMA Netw Open*. Published June 07, 2023. doi:10.1001/jamanetworkopen.2023.17130

Data
Data available: No
